# Multimodal Discrimination between Normal Aging, Mild Cognitive Impairment and Alzheimer’s Disease and Prediction of Cognitive Decline

**DOI:** 10.3390/diagnostics8010014

**Published:** 2018-02-06

**Authors:** Corinna M. Bauer, Howard J. Cabral, Ronald J. Killiany

**Affiliations:** 1 Massachusetts Eye and Ear Infirmary, Department of Ophthalmology, Harvard Medical School, Boston, MA 02114, USA; 2 Department of Biostatistics, Boston University School of Public Health, Boston, MA 02118, USA; hjcab@bu.edu; 3 Department of Environmental Health, Boston University School of Public Health, Boston, MA 02118, USA; killiany@bu.edu; 4 Department of Anatomy and Neurobiology, Center for Biomedical Imaging, Boston University School of Medicine, Boston, MA 02118, USA

**Keywords:** Alzheimer’s disease, mild cognitive impairment, multimodal neuroimaging, cognitive decline

## Abstract

Alzheimer’s Disease (AD) and mild cognitive impairment (MCI) are associated with widespread changes in brain structure and function, as indicated by magnetic resonance imaging (MRI) morphometry and 18-fluorodeoxyglucose position emission tomography (FDG PET) metabolism. Nevertheless, the ability to differentiate between AD, MCI and normal aging groups can be difficult. Thus, the goal of this study was to identify the combination of cerebrospinal fluid (CSF) biomarkers, MRI morphometry, FDG PET metabolism and neuropsychological test scores to that best differentiate between a sample of normal aging subjects and those with MCI and AD from the Alzheimer’s Disease Neuroimaging Initiative. The secondary goal was to determine the neuroimaging variables from MRI, FDG PET and CSF biomarkers that can predict future cognitive decline within each group. To achieve these aims, a series of multivariate stepwise logistic and linear regression models were generated. Combining all neuroimaging modalities and cognitive test scores significantly improved the index of discrimination, especially at the earliest stages of the disease, whereas MRI gray matter morphometry variables best predicted future cognitive decline compared to other neuroimaging variables. Overall these findings demonstrate that a multimodal approach using MRI morphometry, FDG PET metabolism, neuropsychological test scores and CSF biomarkers may provide significantly better discrimination than any modality alone.

## 1. Introduction

Alzheimer’s disease (AD) is the most common form of dementia, currently affecting approximately 5.5 million Americans [1]. Although age is the best-known risk factor for AD [1], the rate of development of AD is heightened in individuals with the amnestic form of mild cognitive impairment (MCI). Amnestic MCI is characterized by cognitive deficits primarily affecting memory with preserved overall cognitive and functional abilities and the absence of a dementia [2]. Individuals with MCI convert to AD at a rate of nearly 8 to 15% per year in comparison to approximately 1% per year in normal aging [2,3,4], making it imperative to generate effective methods for identifying individuals with MCI. There are a number of factors that may contribute to the diagnosis of AD or MCI including performance on neuropsychological tests, brain morphometric measurements, cortical uptake of positron emission tomography (PET) tracers and concentrations of biomarkers in cerebrospinal fluid (CSF).

Pathologically, AD and amnestic MCI are characterized by the presence of intracellular neurofibrillary tangles (NFTs) and extracellular amyloid plaques. The NFTs are composed of insoluble hyperphosphorylated tau protein and reduce the integrity of the cytoskeleton, such that neurons are dysfunctional. Ultimately, this leads to synaptic and neuronal loss [5,6]. Amyloid plaques are extracellular, composed of insoluble fibrils of amyloid-beta (Aβ) and may be related to the hypometabolism that is observed using 18-fluorodeoxyglucose PET (FDG PET) [6]. In AD and MCI, NFTs accumulate in the locus coeruleus, hippocampus, entorhinal cortex, amygdala and other limbic areas that are important for memory [7,8]. As Alzheimer’s dementia progresses, the NFTs affect more neocortical areas, resulting in deficits in other cognitive domains [7,9,10,11]. On the other hand, amyloid plaques tend to accumulate more in the association cortices first and affect hippocampal structures only as the disease progresses [9,12]. Because both NFT density [13] and the extent of amyloid distribution [14] are related to the severity of impaired cognition, it may be possible to monitor the degree of dementia via CSF biomarkers of amyloid and tau pathologies, namely total tau (tTau), hyperphosphorylated tau (pTau) and Aβ-42 [15,16]. These biomarkers are able to identify AD in its early stages with fairly high accuracy [17] and increased levels of pTau and tTau have been observed in AD compared to normal aging [18,19]. Furthermore, CSF samples from both MCI and AD subjects show decreased concentrations of Aβ-42 [19], which may reflect an increased deposition of Aβ in aggregated plaques in the brain [20]. It is evident that increasing concentrations of tau and amyloid in the CSF may be indicative of further progression along the spectrum of Alzheimer’s dementia.

Accumulation of AD pathology can have multiple consequences, including a disruption of synaptic function that may be indirectly measured via changes in glucose metabolism. FDG PET, a glucose analogue, is typically used as a marker of synaptic function, as metabolic changes are closely tied to glucose consumption [21]. There is a relatively consistent pattern of decreased metabolism that occurs in AD. The regions that tend to show hypometabolism are the posterior cingulate/retrosplenial cortex and the cortical structures in the parieto-temporal junction, such as the angular gyrus and precuneus [22,23,24,25,26]. Some studies also indicate a decrease in hippocampal and entorhinal metabolism [6,27,28], although this is not consistently observed. There is a less consistent pattern for MCI [29].

Neuronal loss may also occur as a result of AD pathology. This loss is visible in vivo through MRI changes in cortical surface area, thickness, or volume of the cortical structures. Such morphometric changes have consistently been observed in MCI and AD, with the earliest detectable changes occurring in the entorhinal cortex hippocampus, spreading outward to other cortical and subcortical structures [30,31,32].

Ultimately, the accumulation of pathologies and the resultant changes in synaptic function and neuronal loss manifests as cognitive deficits. Initially, difficulties with memory tasks are often observed, followed by deficits in executive function and ultimately affecting visuospatial abilities and attention in the later stages of the disease [33,34]. The cognitive deficits seen throughout mild cognitive impairment (MCI) and AD correlate with the degree of pathology in post-mortem tissue analysis as well as in vivo imaging measures [5,35,36,37]. However, many of these studies only report one or two tests in the same subjects, thus we do not have a complete picture as to the neural correlates of the wide range of neuropsychological functions in normal aging, MCI and AD.

Each of the four modalities (CSF biomarkers, FDG-PET, MRI morphometry and neuropsychological evaluations) discussed above may be useful for discriminating normal aging, MCI and AD. Since each of the modalities is to an extent independent of the others, it is conceivable that this combination would provide better discrimination than any individual method on its own. In the current study, we explored this concept using a data-driven approach, which may provide additional variables not typically included in an *a priori* analysis. Because each of these factors can provide unique information that can influence diagnosis as a whole, we also examined if the ability to differentiate groups is improved by combining modalities. Furthermore, we sought to determine which neuroimaging variables at baseline were best predictive of future cognitive decline, as measured by annualized percent change (APC) of a battery of standard cognitive tests.

Thus, the main aims were: (1) to determine the best combination of FDG PET, CSF biomarkers, MRI morphometric and neuropsychological test scores for differentiating between normal aging, MCI and AD groups and (2) to identify the MRI morphometric, FDG PET metabolic variables and CSF biomarkers that are able to predict future cognitive decline in normal aging and MCI subjects.

## 2. Materials and Methods

### 2.1. Subjects

Data used in the preparation of this article were obtained from the Alzheimer’s Disease Neuroimaging Initiative (ADNI) database (adni.loni.usc.edu). The ADNI was launched in 2003 as a public-private partnership, led by Principal Investigator Michael W. Weiner. The original arm of ADNI was a 5-year non-randomized natural history non-treatment study utilizing data from multiple study centers across the United States and Canada. The primary goal of ADNI has been to test whether serial magnetic resonance imaging (MRI), positron emission tomography (PET), other biological markers and clinical and neuropsychological assessment can be combined to measure the progression of mild cognitive impairment (MCI) and early Alzheimer’s disease (AD). The data for use in this study were chosen from the larger pool of data that has been made publically available by the Alzheimer’s Disease Neuroimaging Initiative. Data was screened to include all subjects who had both PET and MRI scans available for use on the ADNI website (www.loni.ucla.edu/ADNI) at the time this study began (2008). From this screened dataset, PET data from 21 subjects was of poor contrast and quality and had to be omitted from the analyses undertaken in this study. Three subjects were omitted due to missing information. This left us with data from 403 subjects. We present demographic information on this sample in Table 1.

As part of the ADNI, all subjects completed a battery of neuropsychological tests. On the basis of their cognitive status, the subjects were classified by the ADNI clinical core as: (a) normal controls with normal cognition and memory, Clinical Dementia Rating (CDR) 0 and Mini Mental Status Exam (MMSE) between 24–30; (b) amnestic MCI with memory complaint verified by a study partner, memory loss measured by education-adjusted performance on the Logical Memory II subscale of the Wechsler Memory Scale-Revised [38], preserved activities of daily living, CDR 0.5, MMSE between 24 and 30 and absence of dementia at time of baseline MRI scan; or (c) probable AD with memory complaint validated by an informant, abnormal memory function for age and education level, absence of depression, impaired activities of daily living, diminished cognition, CDR > 0.5 and MMSE between 20–26. For more information about the ADNI please refer to http://www.adni-info.org.

### 2.2. CSF Sampling

Detailed CSF collection and processing methods can be found in elsewhere [39]. Briefly, CSF samples obtained by lumbar puncture were examined for tTau, pTau and Aβ-42 using an immunoassay method. These measures were performed by the ADNI Biomarker Core at the University of Pennsylvania School of Medicine.

### 2.3. Neuropsychological Testing

For this study, we analyzed the cognitive scores from the cognitive and neuropsychological tests taken at the first visit. CDR memory, CDR problem solving and judgment, Trails A, Trails B, Clock draw, Clock copy, digit span forward and backward and the Rey’s Auditory verbal (RAVLT) 30 min delay recognition, 30 min recognition errors and 30 min recall were examined for their ability to differentiate between subject groups in this study.

### 2.4. MRI Processing

For this study, we analyzed the T1-weighted MPRAGE baseline MRI scans from those acquired by the ADNI on 1.5T scanners from General Electric (GE Healthcare, Milwaukee, WI, USA), Philips Medical Systems (Philips, Best, The Netherlands) and Siemens Medical Solutions (Siemens, Erlangen, Germany). Specific pulse sequence guidelines can be found at http://www.loni.ucla.edu/ADNI/Research/Cores/index.shtml.

All MRI and FDG PET scans were processed with the Freesurfer 5.1.0 (Martinos Center for Biomedical Imaging, Boston, MA, USA) [40,41], which is documented and freely available. The processing pipeline has been described in detail elsewhere [40,41,42,43,44,45]. Briefly, for each subject, the 2 DICOM T1-weighted MRI datasets were motion corrected, averaged, segmented into gray matter, white matter and cerebral spinal fluid (CSF) and intensity normalized. The brain was parcellated into cortical and subcortical regions of interest (ROIs) using the Desikan/Killiany atlas [46]. Cortical thickness measures were corrected for gray/white matter intensity ratio (GWIR) using residuals [47]. The gray/white matter intensity ratio was calculated as previously described [48,49]. Briefly, gray matter tissue intensities were measured 35% through the thickness of the cortical ribbon. White matter tissue intensities were measured 1 mm below the gray/white matter boundary, into the white matter. The GWIR was calculated by dividing the white matter by the gray matter intensity values. The ratios were then projected onto the cortical surface and smoothed with a Gaussian kernel with a full width at half maximum of 30 mm.

### 2.5. FDG-PET

For this study, we analyzed baseline FDG-PET scans from those acquired by the ADNI on GE, Philips, or Siemens scanners. Specific protocols for each scanner are available from the ADNI website (http://adni.loni.ucla.edu/research/protocols/pet-protocols/). These data were corrected for radiation attenuation and scatter using scanner-specific algorithms and each image was visually assessed for potential artifacts by the ADNI PET core at the University of Michigan. For this study, we used the original PET data that was not pre-processed by the ADNI PET core so that we could have local control of all the processing steps as with the MRI scans.

The respective PET and MRI images were co-registered using an automated Freesurfer boundary based application [50]. The resulting co-registration was visually assessed for accuracy and adjusted if necessary (approximately 25% of the datasets). Each of the ROIs from the Desikan atlas were reverse transformed into PET space and FDG uptake was calculated in each ROI [46]. A total of 82 cortical and subcortical areas were examined for changes in MRI morphometry and FDG uptake related to MCI and AD relative to normal aging.

To control for individual global variations and to increase sensitivity of the method for differentiating between subject groups [51], the FDG uptake was normalized to regional activity in the cerebellum using residuals [52]. Partial volume effects were also corrected for using an adapted gray matter mask [53].

### 2.6. Statistical Analysis

In order to assess the equality of the male-female distribution in the three diagnostic groups, χ^2^ tests were performed. Age, education and MMSE distributions in the three diagnostic groups were assessed using analysis of variance (ANOVA). Age was correlated with each of the morphometric and uptake variables, including cortical surface area, volume, cortical thickness, gray/white matter intensity ratio and FDG uptake. Hemisphere differences for both MRI and PET data were examined with paired *t*-tests and correlation analysis. All statistical analyses were performed using SAS (SAS Institute Inc., Cary, NC, USA).

In order to determine which neuroimaging variables and neuropsychological tests predicted diagnostic group and future cognitive decline using a data-driven approach, a series of step-wise regression models (logistic and linear, respectively) were created from a total of 282 unique predictors (18 neuropsychological test variables, 17 subcortical volume measures, 68 cortical surface area measures, 68 cortical volume measures, 68 cortical thickness measures, 40 FDG PET variables (averaged between hemispheres) and 3 CSF markers). For both logistic and linear regression models, entry and exit criteria of 0.20 were used. Age, gender and education were forced into the models, effectively controlling for any variance due to these demographic characteristics. To examine the added effects of CSF biomarker concentrations on the multimodal model, we forced all the variables from the multimodal model into the CSF-multimodal model. In this way, we could ensure that the variables contributing variance in the first multimodal model were repeated in the CSF-multimodal model in order to limit the changes in c-statistic to just the CSF biomarker concentrations. In an effort to control for collinearity amongst variables, instances where a ROI was represented by more than one modality in the model (e.g., cortical thickness and FDG uptake), the modality accounting for the most variance in the model was included and the other was excluded. The same process was used to control for models in which both hemispheres were represented from the same modality. For linear regression, standardized estimates of the predictor variables were used, ensuring that they were all in the same scale. Pearson’s correlation was used to assess collinearity amongst the predictor variables of the multimodal models.

Because the logistic regression models are all based on binary outcomes, overall predictive power of the model was determined based on the c-statistic, whereby a value of 1 indicates very high discrimination between groups. Overall goodness of fit was determined based on the Hosmer-Lemeshow method [54]. Because the predicted probabilities obtained from the logistic regression models are used to classify diagnostic groups in this study, the Cox and Snell generalized *R*^2^ with Nagelkerke adjustment [55] was chosen as an additional index of fit. Goodness of fit for the linear regression models was based on percent variance that was explained by the model. In the logistic regression models, the contribution of each variable is determined by the odds ratio, which estimates the change in likelihood of being in one group over the other for a one standard deviation difference in that predictor. For example, in models differentiating AD from MCI and odds ratio below 1 would be associated with increased odds of AD, whereas a value greater than 1 would be more closely associated with MCI. In the linear regression models, the contribution of each variable is determined by the standardized estimate, which provides a measure of the relative importance of the biological variable at predicting the decline in cognitive test measures. The overall model efficacy was determined by the adjusted R-squared.

## 3. Results

Chi-square tests revealed no significant differences for distribution of males and females between groups (df = 2, *p* = 0.3517). Age was not significantly different between control, MCI and AD groups, as indicated by ANOVA (*p* = 0.6684). The AD group had on average one year less education than normal and MCI groups, which, although small, was significant (*p* < 0.05) likely due to the number of subjects. As expected, the MMSE scored also showed significant decreases in both the MCI and AD subject groups (*p* < 0.05).

### 3.1. Left/Right Hemisphere Differences

Volume, cortical thickness and cortical surface area were significantly different between left and right hemispheres in the vast majority of regions, thus data from the hemispheres were analyzed separately for all MRI measures. FDG PET uptake showed no significant differences between hemispheres, so the FDG PET data from the two hemispheres were averaged.

### 3.2. Models for Predicting Diagnostic Group

Separate morphometry, CSF biomarker, FDG PET and neuropsychological test models were generated and the variables contributing unique variance from each of these models were entered into a second level stepwise logistic regression model to generate a multimodal model. Because the presence of CSF measures improved the goodness of fit, only the multimodal models including CSF concentrations of Aβ-1-42, total tau and pTau are reported. Full model details are available in the supplemental material (Tables S1–S20).

### 3.3. Differentiating Groups

The multimodal model containing CSF measures was able to differentiate between AD and MCI well, with a c-statistic of 0.943 (Hosmer-Lemeshow goodness of fit Chi-square = 8.85, *p* = 0.36, Nagelkerke *R*^2^ = 0.70) (Table 2). This increased when differentiating between MCI and normal aging subjects to 0.972 (Hosmer-Lemeshow goodness of fit Chi-square = 3.20, *p* = 0.92, Nagelkerke *R*^2^ = 0.81) (Table 3). When differentiating between AD and normal aging, the c-statistic was 0.998 (Hosmer-Lemeshow goodness of fit Chi-square = 0.33, *p* = 1.0, Nagelkerke *R*^2^ = 0.95). Although CSF concentration of Aβ-1-42 was in the model, it did not contribute significantly to the explained variance (*p* = 0.07) (Table 4). Note that when gender was forced into the model, it became unreliable, with a number of extreme odds ratios for the predictor variables. Thus, gender was not forced into the model for differentiating between AD and normal aging. When differentiating between all three groups the c-statistic was 0.946 (Nagelkerke *R*^2^ = 0.78). The CSF variable included was Aβ-1-42, which was a significant predictor (Table 5).

### 3.4. Cognitive Scores at Baseline and Decline by Diagnostic Group

Baseline scores differed between groups in two main patterns: (1) a stepwise significant decrease between normal aging and MCI and again between MCI and AD (as seen in Trails B, clock score, digit span backward and the RAVLT 30 min delay, delay total and delay errors) and (2) significant decreases in AD but no change between normal aging and MCI (as observed for Trails A and digit span forward). Refer to Table 6 for details.

In order to measure cognitive decline, average annualized percent change measures were calculated for normal aging, MCI and AD groups. Annual percent change showed three main patterns: (1) significantly greater decline in AD compared to both normal and MCI (e.g., clock score, digit span backward and RAVLT 30 min delay), (2) significantly greater decline in AD compared to normal but not MCI (e.g., Trails B and digit span forwards) and (3) no significant differences in the amount of decline between groups (e.g., RAVLT delayed recall and Trails A).

### 3.5. Prediction of Cognitive Decline

A series of linear regression models were created to determine which combinations of neuroimaging and CSF biomarker measures best predicted cognitive decline across clock drawing, trails B, digit span forward and backward and RAVLT delayed recall. Because the goal of the study was to determine neuroimaging predictors of future cognitive decline in the normal and MCI groups, only the models demonstrating poorer performance in these groups will be presented herein. Specifically, decline was only observed in controls for the Trails A and in MCI for RAVLT delayed recall tests.

### 3.6. Longitudinal Changes in Trails A

The average APC for Trails A in normal aging was 0.14 (sd = 0.49), indicating that it took a significantly longer time to complete the test on future visits (*p* = 0.0033). This change was predicted best from combining MRI and FDG PET (*R*^2^ = 0.36, Adj. *R*^2^ = 0.29, *f* = 4.91, *p* < 0.0001). Larger baseline volume in the right temporal pole and surface area in the left banks of the superior temporal sulcus were predictive of greater decline during follow-up. Smaller baseline cortical thickness in the right posterior cingulate, volume in the right thalamus, surface area in the right inferior temporal and hypometabolism of the precentral gyrus were associated with greater decline in Trails A at follow-up, as reflected in the positive APC. The full models can be found in Table 7.

### 3.7. Longitudinal Changes in RAVLT 30 Min Delayed Recall 

The average APC for RAVLT 30 min delayed recall declined in the MCI group over time, with APC values of −0.03 (sd = 0.61), although this did not reach statistical significance (*p* = 0.55). Within the MCI group, combining MRI morphometry and FDG PET metabolism accounted for 26% of the variance (*R*^2^ = 0.31, Adj. *R*^2^ = 0.26, *f* = 6.21, *p* < 0.0001), while combing CSF biomarker concentrations with imaging markers accounted for only 12% of the variance (*R*^2^ = 0.26, Adj. *R*^2^ = 0.12, *f* = 1.85, *p* = 0.07). Older age was significantly predictive of greater change. Larger baseline cortical thickness in the left superior parietal, larger baseline volumes in the left entorhinal, left posterior cingulate and left caudate and larger baseline surface areas in the right postcentral and left pars opercularis were predictive of greater decline in RAVLT 30 min delayed recall. The full models can be found in Table 8.

## 4. Discussion

In this study, we used a data-driven approach to create a set of models that characterize the MRI morphometric, FDG PET, CSF and neuropsychological test variables that are best able to discriminate between normal aging, MCI and AD. We also identified the baseline morphometric, metabolic and CSF biomarker variables associated with cognitive decline in trails and RAVLT delayed recognition in the normal and MCI groups, respectively.

### 4.1. Models for Predicting Normal Aging, MCI and AD

Each modality on its own was able to distinguish between the groups to some degree; however, similar to previous studies, MRI provided a better discrimination than FDG PET [56], CSF biomarker concentration [57], or neuropsychological tests [58]. Furthermore, MRI-calculated volume, cortical thickness and surface area measures were all represented in the model. Given that volume and surface area contributed significantly to the multimodal models predicting diagnostic groups, it may be counterproductive to limit MCI and AD studies to only cortical thickness. Previously, it has been suggested that cortical thickness changes more in AD than cortical surface area when the effects of age are removed [59]. Dickerson et al. [59] failed to observe an effect of AD on cortical surface area in the perirhinal cortex or the parahippocampal gyrus. Our study, on the other hand, showed that there were a number of regions in which cortical surface area was affected by both MCI and AD, even after the effects of age were accounted for. This suggests that surface area may have been unduly overlooked in the past.

Overall, many of the markers identified herein are in agreement with those found previously [58,60,61]. The model differentiating AD from normal aging was relatively simple, with decreased left hippocampal volume being the only imaging predictor. As we attempt to differentiate between earlier stages in disease progression, the models become more complex not only in terms of the number of variables contributing variance but also in that more modalities may be necessary. This is particularly the case for differentiating between MCI and normal aging, in part because MCI represents a broad spectrum with some individuals being more cognitively similar to their typically aging peers and others more similar to AD subjects. Importantly, some key regions that were associated with an increased likelihood of MCI were decreased right hippocampal volume, decreased left caudal middle frontal volume, decreased entorhinal uptake and decreased left entorhinal volume. Histologically, the medial temporal cortex is known to be affected first in the AD trajectory, while the frontal lobe is thought to be relatively preserved until later stages of the disease [9]. Interestingly, decreased volume in a few areas was associated more with normal aging than with CI, including the postcentral gyrus. Age-related decreases in neuronal number within the primary areas for the special senses of the head have been reported previously [62]. It is important then to recognize that there are areas of the brain that may be relatively preserved in the early stages of dementia.

Furthermore, it is plausible that different hemispheres and types of morphometry are affected at different states of the disease. In our multimodal models for differentiating normal aging from MCI, volumes of both the right hippocampus and the left entorhinal cortex were significant predictors, while the left hippocampal volume and right entorhinal cortical thickness were significant predictors for AD vs. MCI. Although the precise neuroanatomical correlates of MRI-derived morphometry measures are not fully characterized, cortical surface area may be linked to brain volume in that it may represent cortical columns, whereas cortical thickness may represent the number of cells within a column [63,64]. It has also been suggested that cortical surface area may be influenced by a variety of factors such as synaptogenesis, dendritic arborization, intracortical myelination and connectivity [65]. Changes in MRI volume are highly correlated with post-mortem measures of tissue volume [66,67,68], which suggests that the volume loss observed in this study likely reflects neuronal loss. Cortical thickness changes are thought to reflect loss of neurons and neuropil. Studies that examine ante-mortem cortical thickness with post-mortem neuron counts show high levels of agreement [45].

#### 4.1.1. Comparison to the Areas Reported as the “Cortical Signature of AD”

Previous studies suggest the entorhinal volume, hippocampus volume [27,69,70], amygdala [69] and inferior temporal lobe volume to be predictive of AD [71]. Other studies also suggest that retrosplenial thickness is able to predict AD [70], while others still rely on what is known as the “cortical signature of AD,” which is a set of 10 cortical thicknesses that have been shown to change consistently in AD [72]. One benefit of the current study to previous studies is that we did not examine only a few preselected regions but rather included the entire cortical and subcortical gray matter. To directly test the benefit of not limiting our data to regions that change most with AD, we created a model that included only the “cortical signature regions,” along with age and education, to see which model differentiated normal aging from AD best. We found that our data-driven approach was better able to differentiate groups, with a significantly larger c-statistic (c = 0.90 for signature regions only and *R* = 0.98 for our model, *p* = 0.0002). In addition, regions typically associated with the signature of Alzheimer’s disease were not all in the models differentiating disease groups, suggesting that although the “Alzheimer’s signature” regions may change most in the disease that they are not optimal for differentiating disease states. Thus, this paper indicates additional brain regions that might be targeted for future studies and perhaps for assisting in clinical diagnosis. Although significant changes were observed throughout the cortex, not all these regions were able to contribute unique and independent variance to the models.

#### 4.1.2. Neuropsychological Tests

There are a number of benefits to using neuropsychological tests for determining diagnostic group, including its low-cost relative to MRI, PET and CSF sampling. There are also no risks to the patient. On the other hand, these tests may not be as specific to differential diagnoses, and they can take a long time to administer and have a high degree of variability. Nonetheless, a number of neuropsychological tests were found to contribute differentially to the ability to discriminate between the various stages of AD progression. For differentiating normal aging from MCI, the earliest stage in the progression, clock drawing, digit span backwards and RAVLT 30 min delayed recall and recognition errors were predictors. In the model differentiating MCI from AD, a mix of visuospatial ability and memory were in the model, as indicated by the presence of Trail A and RAVLT 30 min delayed recall and recognition, while only RAVLT 30 min delayed recall significantly contributed to differentiating AD from normal aging. Taken together, these results show that different combinations of tests were better at differentiating normal aging from MCI than differentiating MCI from AD and normal aging from AD. This is not surprising given the progression of the disease and the basement effects that may be observed in tasks that require more memory and executive function.

#### 4.1.3. CSF Models

We examined which of three biomarkers found in CSF contributed to models differentiating between normal aging, MCI and AD. Aβ-1-42 contributed variance to each of the models. However, this only reached statistical significance for the MCI vs. AD model. For tau measurements, total tau contributed to differentiating between MCI and normal aging and ptau contributed to differentiating between MCI and AD. The ratio of tTau to Aβ-1-42 has been indicated as a unique predictor of diagnostic group previously [70]; however, in a ratio measurement it is unknown whether it is the Aβ-1-42 or the tTau driving the predictive value. tTau and pTau are typically associated with neuronal and axonal damage, while Aβ-1-42 is a reflection of the amyloid burden in the brain. Although CSF measures may be useful in identifying individuals at risk for disease progression, they are not as useful as MRI or neuropsychological tests at differentiating between the groups [71]. This may be in part because the CSF measures are not exclusively brain derived, nor do they provide insight as to the localization of the AD-related pathology.

#### 4.1.4. Role of FDG PET

In this study, we observed very little added benefit for FDG PET scans compared to MRI. This is in agreement with a number of previous studies [56,70,71] and at odds with others that have found evidence for better prediction with FDG PET than with MRI [27,69,73]. While some of these discrepancies may be accounted for by sample, scanner, and scanning protocol, a portion of the difference may be accounted for by differences in post-processing methods. While here we present data from a data-driven ROI-based approach that resampled the PET data into MRI space, many of the other studies use *a priori* ROIs or a voxel-based approach using relatively large voxels, which may be less sensitive to group changes, particularly in small structures or those that may show more anatomical variability. Another post-processing difference lies in the treatment of partial volume effects and normalization region. We controlled for partial volume effects, which diminished some of the group differences [52] and may have contributed to its relatively poor performance compared to MRI morphometric variables. Many of the studies citing an increased ability of FDG PET to detect AD compared to normal aging do not adjust for partial volume errors that occur in PET imaging in atrophic structures, which likely artificially inflates the ability of FDG PET to predict group [69,73,74]. We also normalized to the cerebellum rather than the pons or whole brain based on results of one of our previous studies [52].

### 4.2. Models Predicting Longitudinal Decline in Cognitive Performance

The results of this study indicate that MRI performs better than FDG PET or CSF measures at accounting for variance in the neuropsychological measures at every stage of disease. Combining modalities did not consistently improve the adjusted R^2^ values, nor did FDG PET and CSF alone account for any variance. In many instances, no FDG PET or CSF biomarker concentration variables made it through the initial cutoff stages of building the models. Each of the tests was associated with measures related to widespread regions in the brain, which suggests that each of these tests involves a network of neuronal processing for efficient function. In addition, different regions were typically predictive of baseline performance and decline within each group, and the regions and types of measures varied between groups, illustrating the complex nature of structure-function relationships and the impact of disease upon them.

A number of imaging variables showed opposite relationships than originally anticipated (e.g., larger volumes predicting worse test scores or greater decline). While we are still investigating the exact origins of this negative relationship, one potential explanation is that it represents a compensatory mechanism. This phenomenon is not well understood but has been observed previously [35,75,76,77,78,79,80,81]. The underlying premise being that these brain regions are more associated with a specific task than would normally be the case to help cope with the loss of function in related structures (e.g., the pericalcarine may be compensating for decreased visuospatial processing abilities in other brain regions). Undoubtedly, this may account for some of the inverse relationships that were observed.

### 4.3. Trails A

The neural correlates of Trails A are not well identified and it has not been well characterized on its own in normal aging, MCI and AD, in part because it tends to be used in conjunction with Trails B. We examined both tests individually, rather than taking the ratio of the two, because with ratios, it is unknown whether it is the numerator or denominator that is the driving force behind the relationship. Trails A is thought to reflect abilities in visual scanning, graphomotor and psychomotor speed and attention; as such, we would expect to see associations with the occipital areas, precentral gyrus and regions critical to attention. Baseline FDG uptake in the precentral and postcentral gyri was predictive of APC in the normal aging group, confirming the role of brain regions controlling motor function in Trails A. Attention has also been implicated in Trails A. There are various forms of attention that may be more closely linked with distinct brain regions. Selective attention, whereby attention is focused on a single stimulus while ignoring irrelevant information, is modulated by posterior parietal systems. These areas are important for orienting and shifting attention and may be modulated by basal ganglia structures [82]. According to the Posner model, the intraparietal sulcus/superior parietal lobe and the temporoparietal junction are involved orienting attention to the appropriate location along with the frontal eye fields and inferior frontal gyrus [83,84]. In our normal subjects, Trails A decline was not predicted by the frontal eye fields, which are located in the caudal middle frontal gyrus [85] but rather by the rostral middle frontal and the right pars orbitalis in the inferior frontal gyrus. Thus, our results support the attention component of Trails A.

### 4.4. RAVLT Delayed Recall

Entorhinal associations with declines in recall for the MCI group provide support for the thought that there may be a connection between episodic memory and NFT pathology in the medial temporal lobes. The associations between frontal and parietal regions with declines in recall scores is not surprising, as these regions have been shown to subserve working memory ability [86]. The posterior cingulate is highly interconnected with the medial temporal lobes and has previously been shown to play a role in memory function. In our study, volume of the left posterior cingulate was predictive of declines in recall scores in MCI. In a study examining the correlations between baseline FDG metabolism and subsequent decline in verbal memory in pre-MCI individuals, the posterior cingulate, bilateral parietal and left prefrontal were all correlated with higher rates of decline [87]. Interestingly, in the same study, those who did not decline but remained in the normal aging category at follow-up, showed significant correlations in the posterior and mid-cingulate regions with verbal memory decline [87].

One difficulty in assessing the impact of deficits in recall is that it may represent problems in either learning or in retention, since both would affect the ability to recall information after delays. Although the present study did not separate the results into retention and learning, a previous report in MCI subjects examined high vs. low retainers and learners and observed that both learning and retention were significantly correlated with cortical thickness in the lateral and medial frontal cortex, lateral temporal, medial temporal, anterior temporal, parietal and anterior and posterior cingulate cortices [88]. Meanwhile, retention on its own, after removing the effects of learning, showed correlations with the anterior, medial and ventral temporal lobe, entorhinal, parahippocampus, temporal pole, fusiform and hippocampus [88]. Thus, retention tended to involve more medial structures, while learning was more widespread. In our MCI subjects, we observed more widespread changes, involving temporal as well as frontal and parietal regions, suggesting that as memory deficits progress, difficulties in learning and retention also become more evident. It is also possible that as medial temporal regions become increasingly atrophic they are no longer able to mediate memory function and other brain regions are recruited. This has been observed in functional imaging studies that show the compensatory involvement of a number of regions including the frontal [77,78,89] and cingulate cortices [76,78].

### 4.5. Limitations

There are a few limitations of the present study. The first is that there is a larger proportion of males to females throughout the entire ADNI sample. This is consistent throughout each of the diagnostic groups and was included in each of our models to control for this. Although ADNI collected genetic information on its participants, we did not examine genetic variables, such as ApoE status, which has been shown to influence rate of disease progression in a dose-dependent manner. Also, not all the subjects in our sample had CSF data, which resulted in a smaller sample for the multimodal model including CSF. The predictive models presented herein should also be validated in an independent sample.

There are multiple methods to assess the overall fit of logistic regression statistical models, each of which has its limitations. The pseudo *R*^2^ indices used in this study have been shown to correlate strongly with other pseudo *R*^2^ measures, such as the McFadden [90]. Furthermore, there is no clear consensus as to which method is optimal [91]. Although a comparison of these methods is beyond the scope of the current study, it may be worthwhile to investigate the fit of our models using additional metrics in future studies.

Along the same lines, the current study is limited in that that the model was not evaluated on an independent dataset, which is the gold standard in evaluating model efficacy. The subjects enrolled in ADNI may not be representative of the entire population due to the restrictions on subject enrollment. The MCI and normal aging groups in particular, might be more diverse in the general population. As such, utilizing subject enrolled in later phases of ADNI may not represent a truly independent sample and there are limited large-scale studies that have not only structural MRI but also FDG PET, CSF biomarker collection and a batter of cognitive neuropsychological testing on a sample of healthy aging, MCI and AD participants.

Finally, because all of the data was used to build the model used in this study, it needs to be validated in a separate population to ensure that the predictions being made represent true disease etiology and to eliminate inherent group differences.

## 5. Conclusions

This study shows that combining modalities better differentiates between normal aging and MCI subject groups. It is important to be able to distinguish individuals with MCI as early as possible. By looking outside the typical *a priori* regions, we may improve the ability to identify individuals at risk for developing AD. These individuals should then be followed over a longer period of time to determine who declines in memory and executive function and the brain regions associated with these changes.

In the first portion of this study, a set of MRI, FDG PET, CSF and neuropsychological variables that best differentiates between normal aging, MCI and AD subject groups was determined in a large sample from the ADNI database. In the second part of this study, we generated statistical models for predicting future cognitive decline within normal aging, MCI and AD groups from a number of neuropsychological tests, which each address specific cognitive functions. At baseline, we observed progressively worse scores on neuropsychological tests of visuospatial abilities, attention, executive function, delayed recall, recognition and working memory in MCI and AD. However, over time, the MCI group declined mainly on delayed recall, whereas the normal aging group declined only on Trails A. Overall, the models indicate that MRI was better able to predict future decline than either FDG PET of CSF biomarker concentrations. The brain regions that were associated with each task highlighted the types of cognitive skills required for successful completion of the test and also highlighted that these regions, when damaged, can result in poor memory, executive function and visuospatial abilities.

The results of this study suggest that the imaging and CSF biomarkers most telling of disease severity and decline may be outside the medial temporal lobes and that perhaps it is these other regions, such as the frontal, parietal and cingulate cortices that may be more telling clinical end points.

## Figures and Tables

**Table 1:** Demographic information.

	Subjects (Male/Female)	Age Mean Years (sd)	Education Mean (sd)	MMSE
Normal Aging	105 (64/41)	75.81 (4.75)	15.90 (3.12)	28.98 (1.12)
MCI	204 (137/67)	75.44 (7.22)	15.80 (2.88)	27.15 (1.71) ^a^
AD	94 (56/38)	74.91 (7.37)	14.61 (3.21) ^a,b^	23.48 (2.14) ^a,b^

^a^ significant difference from normal aging (*p* < 0.05), ^b^ significant difference from MCI (*p* < 0.05) (sd = standard deviation, MMSE = mini mental status exam).

**Table 2:** Multimodal model with CSF for differentiating between AD and MCI. The model provided a c-statistic of 0.943.

	Unit	Odds Ratio	Lower CI	Upper CI	*p*-Value
Gender	1.00	0.91	0.24	3.42	0.88
Age	7.27	0.99	0.53	1.85	0.98
Education	3.24	0.48	0.25	0.92	0.03
Left hippocampus volume	545.50	0.81	0.35	1.88	0.62
Left postcentral surface area	446.40	0.74	0.40	1.36	0.33
Right lateral occipital surface area	616.60	0.43	0.21	0.86	0.02
Right entorhinal cortical thickness	0.52	0.37	0.17	0.82	0.01
Left inferior temporal cortical thickness	0.23	0.32	0.13	0.77	0.01
Left insula cortical thickness	0.20	2.48	1.03	5.95	0.04
Clock drawing	1.11	0.65	0.34	1.24	0.19
Digit span backward	2.17	0.30	0.14	0.63	0.001
Trails A	32.68	2.63	1.30	5.32	0.007
RAVLT 30 min delayed recall	2.74	0.31	0.13	0.78	0.01
RAVLT 30 min delayed recognition errors	2.76	2.37	1.28	4.40	0.006
Aβ 1-42	51.47	0.41	0.20	0.86	0.02
Phosphorylated tau	19.52	0.63	0.32	1.24	0.18

RAVLT = Rey Auditory Verbal Learning Test, CI = confidence interval.

**Table 3:** Multimodal model with CSF for differentiating between MCI and normal aging. The model provided a c-statistic of 0.972.

	Unit	Odds Ratio	Lower CI	Upper CI	*p*-Value
Gender	1.00	2.44	0.40	14.79	0.33
Age	6.44	0.52	0.22	1.22	0.13
Education	3.00	1.36	0.61	3.00	0.45
Right hippocampus volume	557.30	0.54	0.19	1.57	0.26
Right caudal anterior cingulate volume	387.00	3.41	1.41	8.23	0.006
Left caudal middle frontal volume	1109.80	0.15	0.05	0.49	0.002
Left entorhinal volume	447.90	0.76	0.34	1.70	0.50
Right postcentral surface area	363.70	0.81	0.36	1.83	0.61
Right inferior temporal surface area	424.50	0.43	0.18	1.03	0.06
Left rostral middle frontal cortical thickness	0.17	0.79	0.16	4.04	0.78
Left medial orbitofrontal cortical thickness	0.20	0.30	0.13	0.72	0.007
Left superior frontal cortical thickness	0.20	4.58	0.99	21.13	0.05
Clock drawing	0.96	0.29	0.12	0.73	0.008
Digit span backward	2.17	0.58	0.26	1.28	0.18
RAVLT 30 min delayed recall	4.02	0.13	0.04	0.43	0.0009
RAVLT 30 min delayed recognition errors	2.32	1.35	0.33	5.59	0.68
Entorhinal FDG	1448.70	0.42	0.19	0.97	0.04
Aβ 1-42	59.92	0.42	0.17	1.03	0.06
Total tau	55.48	2.98	0.94	9.46	0.06

RAVLT = Rey Auditory Verbal Learning Test, CI = confidence interval, FDG = Fluorodeoxyglucose.

**Table 4:** Multimodal model with CSF for differentiating between AD and normal aging. The model provided a c-statistic of 0.998.

	Unit	Odds Ratio	Lower CI	Upper CI	*p*-Value
Age	6.36	0.04	0.002	0.68	0.03
Education	3.46	0.25	0.02	3.82	0.32
Left hippocampus volume	608.80	0.002	<0.001	0.35	0.02
RAVLT 30 min delayed recall	4.42	<0.001	<0.001	0.11	0.01
Aβ 1-42	58.94	0.06	0.003	1.29	0.07

RAVLT = Rey Auditory Verbal Learning Test, CI = confidence interval.

**Table 5:** Multimodal model with CSF for differentiating between all three groups. The model provided a c-statistic of 0.946.

	Unit	Odds Ratio	Lower CI	Upper CI	*p*-Value
Gender	1.00	0.97	0.40	2.33	0.94
Age	6.68	0.69	0.42	1.12	0.14
Education	3.24	0.78	0.51	1.19	0.25
Left hippocampus volume	570.20	0.52	0.28	0.95	0.03
Left caudal middle frontal volume	1079.60	0.65	0.40	1.08	0.09
Right superior parietal cortical thickness	0.19	2.25	0.98	5.16	0.06
Right isthmus of the cingulate cortical thickness	0.26	1.20	0.73	1.97	0.47
Left temporal pole cortical thickness	0.42	1.87	1.10	3.18	0.02
Left postcentral cortical thickness	0.15	0.48	0.25	0.93	0.03
Right entorhinal cortical thickness	0.53	0.53	0.30	0.96	0.03
Left inferior temporal cortical thickness	0.24	0.45	0.24	0.87	0.02
Left medial orbitofrontal cortical thickness	0.20	0.64	0.40	1.03	0.07
Left lateral occipital cortical thickness	0.15	1.62	0.89	2.94	0.11
Right banks STS cortical thickness	0.23	1.12	0.63	1.99	0.70
Right insula cortical thickness	0.22	1.20	0.68	2.14	0.53
Left pars opercularis cortical thickness	0.18	1.29	0.70	2.37	0.41
Right precuneus cortical thickness	0.19	0.63	0.29	1.35	0.24
Clock drawing	1.07	0.65	0.41	1.03	0.06
Digit span forward	1.93	1.03	0.64	1.65	0.91
Digit span backward	2.25	0.51	0.32	0.82	0.005
Trails A	28.21	1.52	0.91	2.54	0.11
RAVLT 30 min delayed recall	3.89	0.31	0.16	0.62	0.0009
RAVLT 30 min delayed recognition errors	2.61	2.10	1.34	3.30	0.001
RAVLT 30 min delayed recognition	3.78	0.77	0.46	1.29	0.32
Isthmus of the cingulate FDG	1921.70	0.55	0.31	0.95	0.03
Postcentral FDG	1549.70	1.22	0.69	2.15	0.49
Pallidum FDG	597.50	1.25	0.78	1.99	0.35
Aβ 1-42	58.13	0.58	0.36	0.91	0.02

RAVLT = Rey Auditory Verbal Learning Test, CI = confidence interval, STS = superior temporal sulcus, FDG = Fluorodeoxyglucose.

**Table 6:** Annual percent change (APC) ANOVA results showing mean (sd) APC from each of the neuropsychological and cognitive tests for normal aging, MCI and AD during follow-up. Tukey’s post-hoc results are presented in the last three columns, whereby a change in letter between groups indicates a significant change.

	df	Mean APC Normal Aging (sd)	Mean APC MCI (sd)	Mean APC AD (sd)	*f*-Value	*p*-Value	Normal Aging	MCI	AD
Clock draw	2	0.11 (0.83)	0.03 (0.59)	−0.28 (0.83)	8.3	0.0003	a	a	b
Digit span forward	2	0.12 (0.55)	0.004 (0.59)	−0.15 (0.98)	3.98	0.02	a	ab	b
Digit span backward	2	0.13 (0.35)	0.04 (0.73)	−0.21 (0.96)	6.21	0.002	a	a	b
Trails A	2	0.14 (0.49)	−0.06 (0.70)	−0.02 (0.72)	3.27	0.04	a	a	a
Trails B	2	−0.06 (0.01)	−0.03 (0.50)	0.15 (1.21)	2.86	0.06	b	ab	a
RAVLT 30 min delayed recall	2	0.14 (0.86)	−0.03 (0.61)	−0.41 (0.38)	7.12	0.001	a	a	b
RAVLT 30 min delayed recognition	2	−0.005 (1.16)	0.05 (0.50)	−0.10 (0.63)	1.25	0.29	a	a	a

RAVLT = Rey Auditory Verbal Learning Test, df = degrees of freedom, APD = annual percent change, sd = standard deviation, AD = Alzheimer’s disease, MCI = mild cognitive impairment.

**Table 7:** Models predicting APC of Trails A in normal aging subjects. The MRI model accounted for 22% of the variance, FDG PET accounted for 8% of the variance and combining modalities accounted for 29% of the variance.

	MRI Model			FDG PET Model			Multimodal Model		
	Parameter Estimate	*p*-Value	Standardized Estimate	Parameter Estimate	*p*-Value	Standardized Estimate	Parameter Estimate	*p*-Value	Standardized Estimate
Age	−0.01	0.29	−0.10	−0.004	0.68	−0.04	−0.01	0.24	−0.11
Gender	0.09	0.37	0.09	0.10	0.36	0.10	0.15	0.13	0.15
Education	0.02	0.32	0.10	0.04	0.02	0.25	0.02	0.20	0.12
Right posterior cingulate cortical thickness	−0.79	0.01	−0.26	--	--	--	−0.66	0.02	−0.21
Right temporal pole volume	0.001	0.002	0.30	--	--	--	0.001	0.001	0.31
Right pars orbitalis volume	−0.0002	0.20	−0.12	--	--	--	−0.0003	0.07	−0.17
Right thalamus volume	−0.0002	0.04	−0.19	--	--	--	−0.0002	0.02	−0.21
Right rostral middle frontal surface area	−0.0001	0.14	−0.14	--	--	--	--	--	--
Right inferior temporal surface area	−0.0003	0.01	−0.25	--	--	--	−0.0003	0.01	−0.24
Left banks STS surface area	0.001	0.01	0.24	--	--	--	0.001	0.01	0.26
Postcentral FDG PET	--	--	--	0.0001	0.11	0.39	--	--	--
Precentral FDG PET	--	--	--	−0.0002	0.01	−0.62	−0.0001	0.001	−0.31

FDG PET = fluorodeoxyglucose positron emission tomography.

**Table 8:** Models predicting APC of RAVLT 30 min delayed recall in MCI subjects. The MRI model accounted for 26% of the variance, FDG PET accounted for 1% of the variance and CSF accounted for 1% of the variance. Combining imaging modalities accounted for 26% of the variance, while combining all modalities accounted for 12% of the variance.

	MRI Model			FDG PET Model			CSF Model			Multimodal Imaging Model			Multimodal Model		
	Parameter Estimate	*p*-Value	Standardized Estimate	Parameter Estimate	*p*-Value	Standardized Estimate	Parameter Estimate	*p*-Value	Standardized Estimate	Parameter Estimate	*p*-Value	Standardized Estimate	Parameter Estimate	*p*-Value	Standardized Estimate
Age	0.02	0.002	0.27	0.004	0.60	0.05	0.005	0.59	0.07	0.02	0.002	0.27	0.02	0.06	0.29
Gender	−0.09	0.36	−0.07	−0.14	0.25	−0.10	−0.08	0.58	−0.07	−0.08	0.42	−0.06	−0.09	0.54	−0.08
Education	−0.02	0.31	−0.08	−0.02	0.40	−0.07	0.01	0.58	0.07	−0.02	0.21	−0.10	0.01	0.50	0.08
Left superior parietal cortical thickness	0.60	0.02	0.19	--	--	--	--	--	--	0.55	0.04	0.17	0.29	0.39	0.12
Left entorhinal volume	0.0003	0.01	0.21	--	--	--	--	--	--	0.0003	0.01	0.22	0.0003	0.09	0.22
Left posterior cingulate volume	0.0003	0.02	0.19	--	--	--	--	--	--	0.0003	0.04	0.17	0.0003	0.07	0.24
Left caudate volume	0.0002	0.03	0.17	--	--	--	--	--	--	0.0002	0.03	0.18	0.0003	0.13	0.19
Right postcentral surface area	0.0004	0.0002	0.29	--	--	--	--	--	--	0.0004	0.0003	0.29	0.0001	0.48	0.09
Left pars opercularis surface area	0.0005	0.002	0.24	--	--	--	--	--	--	0.001	0.003	0.24	0.0005	0.07	0.24
Postcentral FDG PET	--	--	--	−0.0001	0.16	−0.13	--	--	--	--	--	--	--	--	--
Phosphorylated tau	--	--	--	--	--	--	−0.01	0.06	−0.24	--	--	--	−0.01	0.16	−0.18

FDG PET = fluorodeoxyglucose positron emission tomography, CSF = cerebrospinal fluid.

## Supplementary Materials

Supplementary materials can be found at http://www.mdpi.com/2075-4418/8/1/14/s1.
